# Shipping and the Paris climate agreement: a focus on committed emissions

**DOI:** 10.1186/s42500-020-00015-2

**Published:** 2020-06-12

**Authors:** Simon Bullock, James Mason, John Broderick, Alice Larkin

**Affiliations:** Tyndall Centre for Climate Change Research, University of Manchester, Manchester, UK

## Abstract

The concept of “committed emissions” allows us to understand what proportion of the Paris-constrained and rapidly diminishing global carbon dioxide (CO_2_) budget is potentially taken up by existing infrastructure. Here, this concept is applied to international shipping, where long-lived assets increase the likelihood for high levels of committed emissions. To date, committed emissions studies have focussed predominantly on the power sector, or on global analyses in which shipping is a small element, with assumptions of asset lifetimes extrapolated from other transport modes. This study analyses new CO_2_, ship age and scrappage datasets covering the 11,000 ships included in the European Union’s new emissions monitoring scheme (EU MRV), to deliver original insights on the speed at which new and existing shipping infrastructure must be decarbonised. These results, using ship-specific assumptions on asset lifetimes, show higher committed emissions for shipping than previous estimates based on asset lifetimes similar to the road transport sector. The estimated baseline committed emissions value is equivalent to 85–212% of the carbon budget for 1.5 °C that is available for these EU MRV ships, with the central case exceeding the available carbon budget. The sector does, however, have significant potential to reduce this committed emissions figure without premature scrappage through a combination of slow speeds, operational and technical efficiency measures, and the timely retrofitting of ships to use zero-carbon fuels. Here, it is shown that if mitigation measures are applied comprehensively through strong and rapid policy implementation in the 2020s, and if zero-carbon ships are deployed rapidly from 2030, it is still possible for the ships in the EU MRV system to stay within 1.5 °C carbon budgets. Alongside this, as there are wide variations between and within ship types, this new analysis sheds light on opportunities for decision-makers to tailor policy interventions to deliver more effective CO_2_ mitigation. Delays to appropriately stringent policy implementation would mean additional measures, such as premature scrappage or curbing the growth in shipping tonne-km, become necessary to meet the Paris climate goals.

## Introduction

### Climate change and committed emissions

The UNFCCC Paris Agreement sets out globally agreed goals for action on climate change, aiming to keep the global surface temperature rise well below 2 °C above pre-industrial levels, while pursuing efforts to keep below 1.5 °C [1].

The global climate responds approximately linearly to cumulative carbon dioxide (CO_2_) emissions, and over the timeframes of relevance to the Paris Agreement goals, the rise in global mean surface temperature is strongly dependent on cumulative emissions of CO_2_ [2]. As such, cumulative CO_2_ emissions to 2100 are a better predictor of climate stabilisation than rates of change in emissions, concentration targets and emission levels in a given year [3]. A carbon budget is the quantity of cumulative CO_2_ that can be emitted over time to deliver a prescribed probability of staying below a given temperature target. This carbon budget metric has been used in national and global mitigation studies [4–7] and incorporated as a core concept in IPCC reports [8, 9]. Measures to reduce other greenhouse gas emissions such as methane are also required to achieve the Paris Agreement goals. Different gases have different lifetimes and warming effects so the climate’s response to non-CO_2_ emissions are modelled independently of cumulative CO_2_ [8].

Long-lived fossil-fuel infrastructure assets are prone to “carbon lock-in” [10], committing sectors and economies to CO_2_ emissions years and often decades into the future [11]. The concept of committed emissions from existing infrastructure has been used to examine what proportion of carbon budgets might be taken-up by the future operation of existing high-carbon assets [12, 13]. At a global level, Tong et al. [13]‘s study of committed emissions concludes that “*little or no additional CO*_*2*_*-emitting infrastructure can be commissioned*” and also that early retirement of existing high-carbon infrastructure might be required to meet the limits laid out in the Paris Agreement.

### Committed emissions and shipping

The shipping sector is vital to the world’s economy – it transports over 80% of the world’s trade by volume [14]. However, it is also a major contributor of greenhouse gas emissions, with international shipping emitting around 800 MtCO_2_ a year [15, 16]. If the sector were a country, it would be the 6th highest emitter in the world, ranked between Germany and Japan. As such, the shipping sector needs to make substantial cuts in emissions to play its part in meeting the Paris Climate Agreement goals.

In addition to measures implemented in 2013 to improve efficiency through the design of new ships [17], the international maritime sector has set climate change targets, of an at least 50% reduction in greenhouse gas emissions by 2050 versus 2008 levels [18]. Ships have a long-lifespan, the average age of a ship scrapped in 2018 was 28 years [19], and so these targets may be harder to achieve than in sectors with a more rapid turn-over of assets. There is also the potential for committed emissions from existing ships to take up a high percentage of any carbon budget ascribed to the shipping sector. Analysis of committed emissions from existing shipping assets can therefore inform the rate, extent and types of response required from the shipping sector, for both existing and future ships, towards meeting the Paris climate goals.

**Fig. 1:**
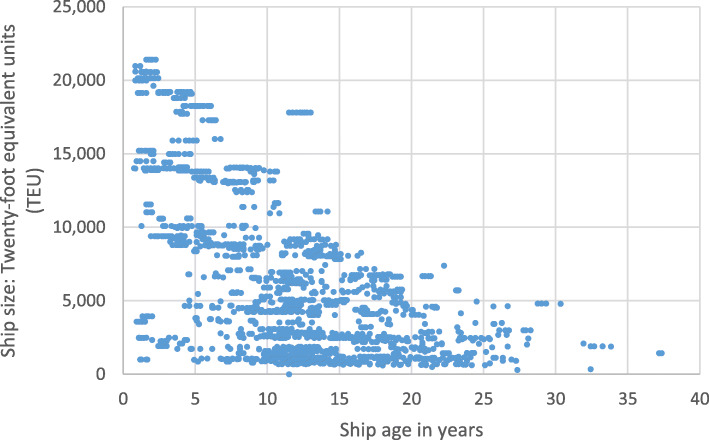
Comparison of ship size versus age for container ships in the EU MRV. Ship size given in TEUs, “twenty-foot equivalent units”, a standard shipping container. Source: authors’ analysis of 2019 fleet data from Clarksons [24]
iii. Unlike for example in aviation, many of the proposed solutions for lowering emissions in the shipping sector can be applied to the existing fleet [25], not just to new assets. A committed emissions figure in the shipping sector should take into the account the potential for existing assets to produce less CO_2_ in future.

To date, studies of committed emissions have been in-depth analysis of the power sector [20–22] or high-level global analyses where shipping is one of many sectors considered [12, 13, 23]. To estimate shipping emissions, global-coverage papers have taken assumptions from elsewhere in transportation. Smith et al. [23] assume asset lifetimes for ships to be similar to those for aviation, and Tong et al. [13] employ the assumptions used in Davis et al. [12], that shipping and aviation assets would have similar lifetimes as those in the road transport sector at 17–28 years. However, there are three reasons, explored in detail in this paper, why a more in-depth analysis for the shipping sector would significantly augment these global analyses:

i. Assuming lifetimes similar to the road transport sector under-estimates committed emissions in shipping, as typically ships have an average scrappage age of 28 years, higher than road transport averages.
ii. The size distribution, age distribution and average age at scrappage of existing assets in the shipping sector varies considerably, both between different ship types and by size within type. A more granular analysis better accounts for such differences than use of sector-wide averages. An example of a within-type difference is set out in Fig. 1 using data for container vessels in the EU MRV system, with many newer ships being over four times larger than those 20 years or older.

This then points to a need to evaluate the committed emissions of the shipping sector in greater detail, to reflect the specific nature of the sector. This paper is the first to analyse committed emissions at the level of individual ships, using new datasets published for the first time in mid-2019. This paper presents an evaluation of a large subset of global shipping’s committed emissions under various assumptions, and sets out implications for efforts to mitigate shipping CO_2_ in line with global climate change goals.

The methods section presents the datasets used, issues concerning data quality, and details for how committed emissions is calculated, under different assumptions. The results and discussion sections identify the committed emissions across ship types for existing ships and for new ships, and compare the range of total committed emissions with a range of different carbon budgets for the shipping sector. They assess which measures might be more important in delivering rapid mitigation in each ship type, given the different age and committed emissions profiles of these sub-fleets, and whether such mitigation measures could bring emissions within carbon budgets. The conclusions section sets out implications for the challenges facing shipping in tackling climate change. Further detail on methodology can be found in supplementary information and the accompanying spreadsheet.

## Methods

This section is split into five parts. First, a description of the datasets related to shipping CO_2_ emissions; second, a methodology for calculating the baseline committed emissions figure for existing ships in a segment of the global fleet; third, methods for assessing how this baseline value might change under different assumptions; fourth, a comparison of this range of committed emissions with a range of possible carbon budgets for this shipping segment, and; fifth, an assessment of factors affecting potential emissions from ships which will be built in the next decade. This section is abridged; further details can be found in supplementary information.

### Data

The shipping sector’s global greenhouse gas emissions have been analysed at an aggregate level by the IMO [16], and in other studies [15]. However, until recently data has not been freely available to assess ship-level greenhouse gas emissions. Data released in July 2019 from the European Union’s new Monitoring, Reporting and Verification (EU MRV) system for CO_2_ emissions allows this granularity [26]. The International Maritime Organisation’s Data Collection System (IMO DCS) commenced in 2019 with global coverage but individual ship data will be kept confidential [27].

The EU MRV system’s initial reporting period was for 2018, with publicly available ship-level data for fuel consumption, energy efficiency and CO_2_ emissions for approximately 11,000 ships. Data is updated regularly; this paper is based on data published during September 2019. To date, one published article has assessed this new EU database – analysing energy efficiency data for a subset of the bulk carrier fleet [28]. We present the first analysis of the CO_2_ data across the whole of the EU MRV dataset, and combine this data with other datasets to provide a new analysis of how these CO_2_ data vary by ship age across all ship types. This first evaluation of data on CO_2_ emissions versus age allows the most detailed estimation to date on committed emissions from a large sub-section of global shipping.

Two other data sets are also used; Clarksons World Fleet Register [24] datasets by ship type for IMO number, size (e.g. deadweight tonnage (DWT)) and age, and Clarksons World Shipyard Monitor [19] for average scrapping age of each type and size of ship worldwide. The individual ship data from Clarksons is provided on a commercial basis and cannot be disclosed, however aggregate results are presented in this paper. Combined, these datasets give a value for expected years of remaining service-life for each of the 11,000 ships.

The EU MRV regulation covers all ships over 5000 gross tonnes, with some limited exemptions (e.g. warships), with coverage of over 90% of CO_2_ emissions from ships calling at EU ports, in 15 ship categories [26]. It requires reporting of the annual CO_2_ emissions from all ships’ journeys that include an EU member state port as destination or departure point, and also emissions at berth in EU ports. It includes emissions from journeys involving non-EU ports: so long as at least one of the destination or departure ports is in the EU. The coverage is therefore not of ships registered in the EU, but ships’ journeys involving an EU port. The ship-owner must also report on fuel types, emissions factors, ship energy efficiency, transport work, total distance travelled and time at sea. The EU’s reporting system uses recorded fuel consumption as the main basis for its calculations. Data quality is good, with minor issues discussed in supplementary information.

### Baseline committed emissions

A baseline committed emissions value for each ship is calculated, by multiplying each ship’s remaining service life by its current annual emissions. A ship’s remaining service life is calculated by subtracting its likely scrappage age from its current age. For each ship its scrappage age is estimated by taking the values of average scrappage age for ships of its type and size, for each year in the last 10 years’ publications of Clarkson’s World Shipyard Monitor, and taking an average value across these 10 years. It is assumed that in future years until scrappage, a ship’s annual emissions are the same as in 2018. See Supplementary information for details and sensitivity analyses on these assumptions.

The total baseline emissions from each ship class, $E{(i)}_{baseline}^{class}$, is calculated by summing across the total number of ships in each class, *N*_*T*_, where *i* represents each individual ship. The total baseline emissions from the full fleet, ${E}_{baseline}^{fleet}$, is then calculated by summing across the total number of classes, *N*_*class*_.

1 $$ E{(i)}_{baseline}^{ship}=\left[t{(i)}_{ship\  age}-{t}_{scrappage}\right]\ast E{(i)}_{annual}^{ship} $$

2 $$ E{(i)}_{baseline}^{class}=\sum \limits_{i=1}^{N_T}E{(i)}_{baseline}^{ship} $$

3 $$ E{(i)}_{baseline}^{fleet}=\sum \limits_{class=1}^{N_{class}}E{(i)}_{baseline}^{class} $$

### Mitigation measures that may alter baseline committed emissions

There are a wide range of options for cutting CO_2_ emissions from shipping [29]. A literature review by Bouman et al. [30] summarises some of these options, in five categories, see Table 1.

**Table 1:** Measures to cut shipping CO_2_ emissions

Category	Measure
Hull Design	Vessel size, hull shape, light-weight materials, air lubrication, resistance-reduction devices, ballast water reduction, hull coating
Power and propulsion system	Hybrid power, power system machinery, propulsion efficiency devices, waste heat recovery, on-board power demand
Alternative fuels	Biofuels, Liquefied Natural Gas (LNG)
Alternative energy sources	Wind-power, fuel cells, cold ironing, solar power
Operation	Speed optimisation, capacity utilization, voyage optimisation, other operational measures

Source**:** Based on Bouman et al. [30]

This list is not exhaustive, for example ammonia, hydrogen and batteries could be added to the alternative fuels category. Implementing any or all of these various options would lower the total value for committed emissions. Similarly, external changes in demand or the organisation of production-consumption networks that use the shipping sector, either due to ongoing economic development or deliberate policy intervention, also have substantial potential to reduce emissions [31].

It is also uncertain to what extent the identified improvements would be applied in future, both at total fleet level, or in ship types, and in different regions, given multiple uncertainties around cost, demand, regulation, and technological deployment rates. To explore how these interventions might alter the committed emissions, a model is constructed to allow user inputs to vary the start times, diffusion rate and deployment levels for different classes of improvement for all ships in each ship class. Four inputs are applied sequentially to the baseline:

- Lower speeds: reducing overall energy required;
- Technical and operational efficiency measures: reducing the fuel required for ships’ propulsion, use of shore-power for ships in port (“cold-ironing”), etc.;
- Blending-in quantities of zero-carbon fuels: reducing the carbon intensity of fuel used;
- Zero-carbon retrofits: full-conversion to zero-carbon fuels.

There are challenges associated with combining the various values for CO_2_ reduction potential published in the literature for use in a simple model. These include: large ranges of uncertainty; the extent to which individual measures are additive; varying impact between ships of different type, age and size; and uncertain impacts of economic and political factors on the extent of technological uptake. In this study, given the large uncertainties in future deployment of the various options, “low”, “mid” and “high” ranges for each type of measure are used, as set out in Table 2. The details of the assumptions and literature sources used as the basis for Table 2 are set out in supplementary information.

**Table 2:** Model input assumptions for calculating reductions to baseline committed emissions

Measure	Input value	Low	Mid	High
Speed	Slow speed improvement factor	0.85	0.75	0.65
	Year slow speed improvement starts	2030	2024	2022
	Years until total speed improvement achieved	10	5	3
Technical and operational	Non-speed: improvement factor	0.95	0.80	0.65
	Non-speed: years until improvements achieved	15	10	8
Blending	Blending: year starts	2030	2025	2022
	Additional annual % of ships using blended fuel	1	2	4
	Additional annual rise in % blended fuel = zero C	1	2	4
Zero-carbon fuel	Year zero-carbon fuel available for retrofit	2035	2030	2025
	Years till all ships retrofitted to use zero C fuel	15	10	10

This paper focusses on reducing on-ship CO_2_ emissions, however meeting the Paris climate goals requires consideration of full life-cycle emissions, of CO_2_ and other greenhouse gases. Whilst LNG is conspicuous for its high life-cycle methane emissions [32, 33], the potential benefits of other alternative fuels are also highly dependent on the balance of CO_2_ upstream and non-CO2 emissions in production, distribution and use [34, 35]. This paper assumes that LNG is not a sufficiently low carbon solution for shipping. We accept that in practice it will be difficult to achieve genuinely zero carbon fuels, and use the term “zero-carbon fuel” to mean fuels which have close to net-zero whole life-cycle greenhouse gas emissions, for example liquid hydrogen produced by hydrolysis of water using wind power.

Whilst our analysis has focussed on technological and operational measures to cut emissions; a further set of policy options would be measures to reduce the volume of goods and the distances they are transported. It may be the case that in future these options are raised up the political agenda, as decarbonisation imperatives grow with increasing climate impacts, or if technological and operational measures are slow in delivery. The potential impact of stronger decarbonisation policy on shipped trade is discussed in depth by Walsh et al. 2019 [36]. For this analysis, our starting assumption is that global climate mitigation effects on volumes of shipped trade would be to lower trade growth, rather than lead to absolute reductions, following the Low Energy Demand scenario included in IPCC SR1.5 [37].

### Shipping carbon budgets

The concept of carbon budgets is prominent in IPCC reports [8, 9], given the near linear relationship between cumulative carbon dioxide emissions and temperature rise. For ships covered by the EU MRV system, we calculate carbon budgets for meeting the Paris Agreement’s goal to “pursue efforts” to keep warming to below 1.5 °C in two stages. First, we set out a range of global carbon budgets to indicate equivalence with this goal, then we ascribe a proportion of that global carbon budget to ships in the EU MRV system.

This paper takes as its starting point the carbon budgets set out in IPCC SR 1.5 [38]. Work since then is summarised in Rogelj et al. [39] who propose a framework for consolidating the various carbon budget methodologies and uncertainties, and sets out the remaining (2019 onwards) global carbon budget for 33, 50 and 66% probabilities of staying under 1.5 °C warming, at 700, 440 and 280 GtCO_2_ respectively. Rogelj et al. also ascribe a variation to these budgets of ± 250 GtCO_2_ to account for uncertainty in the success of policies to mitigate non-CO_2_ emissions such as methane. The complex outlook for non-CO_2_ mitigation is summarised in depth by Roe et al. [40].

In applying a budgeting approach to shipping specifically, we acknowledge that there is no established or agreed mechanism for ascribing a share of global carbon budgets to this sector. Typically, the literature on apportionment methodologies in shipping has focused on the issue of dividing effort between nations [41–44], given the complexities of ownership, operation and use, rather than determining a share of emissions for the shipping sector as a whole. Traut et al. [45] propose that the share of a future global carbon budget for international shipping should be “proportionate to the sector’s current share of global emissions”, an approach which has also been articulated by the International Chamber of Shipping [46]. There are, however, arguments that shipping should receive a larger share, given its vital role in facilitating global trade [47], and likewise arguments the sector should receive less, for example given its greater capability to make emissions reductions than sectors such as aviation or agriculture [25, 29].

We adopt the “proportionate to current share” approach for EU MRV shipping, assuming that the ratio of EU MRV carbon budget to global carbon budget is the same as the ratio of 2018 EU MRV CO_2_ emissions to 2018 Global CO_2_ emissions. We note that taking this approach might overestimate EU MRV budgets, as equity methodologies in the mitigation literature tend to focus on capability and responsibility, and by most metrics the EU has more capability and responsibility than the global average [48]. Nevertheless, sensitivity tests on 10% higher and lower budgets for both international shipping and EU MRV shipping produced marginal difference in implications for the sector (see results section).

### Committed emissions from future ships

Finally, in addition to committed emissions from existing ships, there are also committed emissions from ships to be built in coming years. For example, there are roughly 3000 ships on global orderbooks for delivery over the next 3 years, which will use high-carbon fuels, and be used on average for over two decades. Beyond this, as existing ships are scrapped and replaced, and if the global ship fleet size continues to increase as per historic trends, then these new ships (replacement and additional) will also contribute further emissions, given that it is not likely that a large proportion of new ships will run on zero-carbon fuels until at least 2030 without significant policy changes [49]. This paper briefly discusses the likely relative size of the emissions of existing versus new ships, and implications for climate change policy in the shipping sector.

Fleet growth is highly uncertain. DNV-GL assume that global fleet dead-weight tonnage will increase by 35% on 2016 levels by 2050 [49]. However, this may not translate into shipping tonne mile growth; for example de Backer and Flaig [50] argue that digitalisation and use of robotics may increase intra-regional trade at the expense of inter-regional trade. Furthermore, growth in different fleet sub-categories will vary. DNV-GL’s maritime outlook forecasts that global growth is strong for most sectors to 2030, but from 2030 oil tanker trade will fall. Falling demand for transportation of other fossil-fuels may also have implications for shipping [31, 51]. In this paper we analyse 1, 2 and 3% annual growth rates in the total number of ships, applied uniformly across the whole fleet. We also tested this assumption against ship category variations, for example keeping the overall fleet growth rate at 2% per annum but reducing oil tanker growth to 1% reduces overall new ship committed emissions by 4%. We also note the extremely uncertain long-term impact of the ongoing COVID-19 pandemic, both on shipping generally and on specific sub-sectors such as cruise. In the short-term there will be major effects, although it is unknown what the long-term impacts will be. For instance, following the 2008 financial down-turn the shipping industry rebounded quickly, with tonne-miles transported 10 years later aligning with prior trends [52].

Ships are becoming more fuel-efficient every year driven by measures such as the Energy Efficiency Design Index (EEDI) [53]. Analysis of IMO data by Transport and Environment [53, 54] shows that current new ships are considerably more fuel-efficient than existing ships. The top 10% performing new ships are between 40 and 60% more fuel efficient than comparable ships of the same type and size. In this analysis we assume as an initial baseline that new ships are 40% more energy-efficient than existing ships of the same type and size, with a further annual 3% improvement in EEDI values.

## Results

### Existing ships’ emissions in 2018

The EU MRV database splits ships into 15 categories. Table 3 sets out the total CO_2_ emissions and average emission per ship, in each ship type.

**Table 3:** 2018 CO_2_ emissions by ship type; EU MRV data

Ship type	Number of ships with fuel and CO_2_ data	Total fuel/year (Mt)	Total CO_2/_year (MtCO_2_)	Average CO_2_ /ship/year (tCO_2_)
Bulk Carriers	3311	5.57	17.46	5272
Chemical Tankers	1268	2.91	9.13	7199
Combination Carriers	7	0.03	0.08	12,013
Container Ships	1665	14.04	44.07	26,467
Container Ro-Ro Cargo	72	0.46	1.43	19,890
Gas Carriers	294	0.79	2.45	8340
General Cargo Ships	1048	1.87	5.88	5612
LNG Carriers	194	1.90	5.46	28,154
Oil Tankers	1686	5.62	17.67	10,479
Other Ship Types	104	0.33	1.03	9933
Passenger Ships	146	2.03	6.39	43,776
Refrigerated Cargo	140	0.57	1.78	12,730
Ro Pax	344	4.30	13.78	40,060
Ro-Ro	257	1.89	5.91	23,009
Vehicle Carriers	433	1.62	5.07	11,702
**TOTAL**	10,966	43.94	137.65	12,553

Source: EU MRV database. NB: EU “passenger ship” definition equates to “cruise ships” in Clarksons

*MtCO*_*2*_ million tonnes of carbon dioxide

2018 EU MRV CO_2_ emissions total 138 MtCO_2_, around 17% of international shipping CO_2_ [15]. 67% of these emissions comes from 4 ship types – container vessels, bulk carriers, oil tankers, and roll-on roll-off passenger ships (“ro pax”).

### Baseline committed emissions

**Fig. 2:**
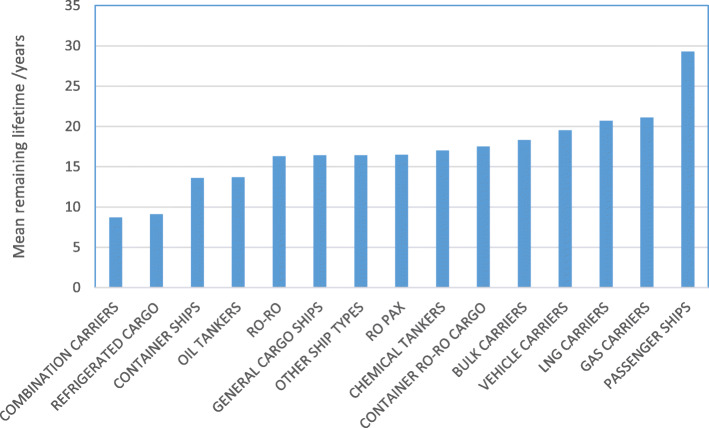
Mean remaining lifetime, by ship type

There is a large variation in the average remaining life for ships of each type, shown in Fig. 2. Values for remaining ship service life and CO_2_ emissions per ship allows calculation of a baseline committed emissions figure for each ship, and for the full EU MRV fleet, as set out above in Eqs. 1–3.

**Fig. 3:**
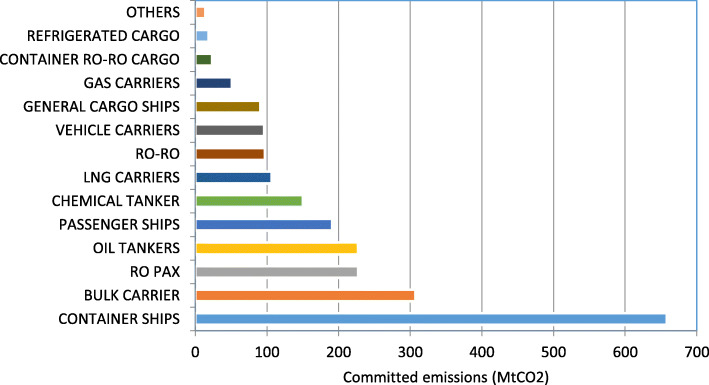
Baseline committed emissions 2019–2050, by ship type, in MtCO_2_

These results from the model for individual ship baseline committed emissions are summed over ship type, and shown in Table 4 and Fig. 3. The headline figure from Table 4, and the central result of this paper, is that the baseline committed emissions from all existing ships in the EU MRV fleet is 2260 MtCO_2_.

**Table 4:** Baseline committed emissions for existing ships, by ship type

Ship type	Number of ships with fuel/CO_2_ data	Total CO_2/_yr (Mt CO_2_)	Committed future CO_2_ (MtCO_2_)	Ratio of committed to current	Average age	Scrappage age + assumption
Bulk Carrier	3311	17.5	307	17.6	8.7	Varies by size: 21.6–32.3
Chemical Tanker	1268	9.1	150	16.5	9.8	26.8
Combination Carriers	7	0.1	1	9.3	15.6	24.3
Container Ships	1665	44.1	658	14.9	11.7	Varies by size: 19.7–28.2
Container Ro-Ro Cargo	72	1.4	24	16.5	12.1	28.2
Gas Carriers	294	2.5	51	20.9	8.5	29.4
General Cargo Ships	1048	5.9	91	15.4	12.5	28.2
LNG Carriers	194	5.5	107	19.5	8.7	29.4
Oil Tankers	1686	17.7	227	12.8	9.7	Varies by type/size: 20.0–30.8
Other Ship Types	104	1.0	13	12.6	17.6	28.7
Passenger Ships	146	6.4	191	30.0	17.2	44.5
Refrigerated Cargo	140	1.8	19	10.6	22.3	30.8
Ro Pax	344	13.8	228	16.5	22.5	Varies by type: 30.8–44.5
Ro-Ro	257	5.9	97	16.5	15.8	30.8
Vehicle Carriers	433	5.1	96	19.0	11.6	30.8
**TOTAL**	10,966	137.7	2260	16.4	10.8

**Fig. 4:**
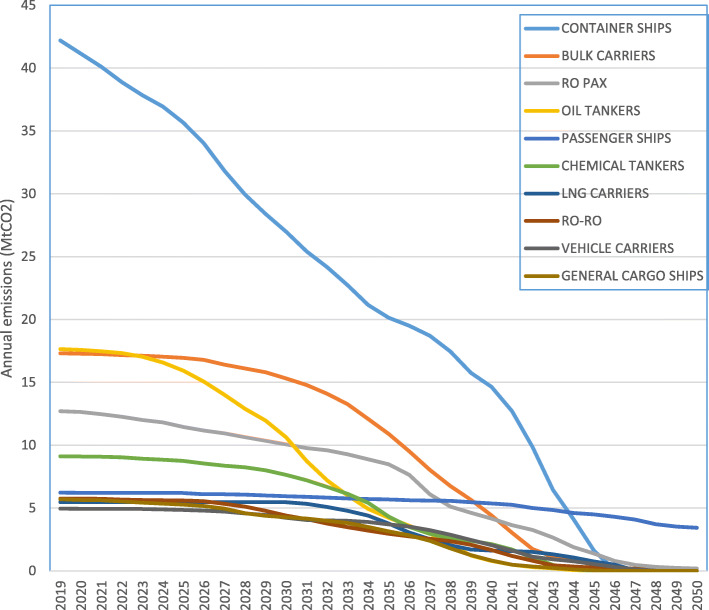
Committed emissions for existing ships, from the 10 largest ship types (by emissions total) through time. Total committed emissions for a ship type are represented by the area under its curve

Five ship types account for 71% of baseline committed emissions: container, bulk carriers, ro pax, oil tankers and passenger ships. The split of cumulative committed emissions in MtCO_2_ for the ten largest ship types is shown in Fig. 4.

The baseline committed emissions data show distinct differences between comparable ship types:

- **Passenger ships and ro pax**

The average ro pax ship is 5 years older than the average passenger (cruise) ship. Also, passenger ships are on average older when they are scrapped. Both factors act to give passenger ships a greater average remaining life. Consequently, the total committed emissions from passenger ships is only 16% lower than for ro pax ships, despite there being 58% fewer passenger ships.

- **Gas carriers and refrigerated cargo ships**

Gas carriers and refrigerated cargo ships (“reefers”) have similar average lifetimes, but the gas carrier fleet is much younger on average (8.5 vs 22.3 years) with far longer expected remaining life. This, plus the greater numbers of gas carriers, counterbalances gas carrier ships having lower average annual emissions than reefers. Overall, the gas carrier ship type has higher baseline committed emissions (51 MtCO_2_ vs 19 MtCO_2_).

Matching the age data with ship size data also highlights variations within ship types. For example, within the container ship type it is newer container ships that are responsible for the highest committed emissions. Despite newer container ships being much more efficient, the recent trend for container ships to become much larger has a greater impact. Graphs showing these trends are available in supplementary information (Figures S3-S5).

### Comparison with shipping carbon budgets

The total CO_2_ emitted in 2018 from the 10,966 ships submitting 2018 CO_2_ data to the EU MRV system is 137.7 MtCO2. Global CO_2_ emissions in 2018 were 36.6 GtCO_2_ [55]. Using the Traut et al. [45] assumptions, the carbon budget for the ships in the EU MRV system is proportionate to their share of total global emissions at 0.38%.

As set out in the methods section, this paper uses a range of carbon budgets expressing different probabilities of meeting the Paris Agreement’s 1.5 °C goal. The share of the remaining global carbon budget for ships in the EU MRV system for these probabilities of keeping below 1.5 °C warming is set out in Table 5, and compared with the baseline committed emissions from existing ships in the EU MRV of 2260 MtCO_2_.

**Table 5:** EU and Global Carbon budgets for different probabilities of meeting 1.5 °C goal

GOAL	Global Carbon Budget (MtCO_2_)	1.5 °C Carbon Budget For ships in EU MRV (MtCO_2_)	EU MRV baseline committed emissions (MtCO_2_)	EU MRV baseline committed emissions as a % of Global Carbon Budget
< 1.5 °C 33% probability	700,000	2650	2260	85%
< 1.5 °C 50% probability	440,000	1670	2260	135%
< 1.5 °C 66% probability	280,000	1070	2260	212%

### Carbon budgets implied by IMO targets

The IMO currently has a strategy and target to reduce greenhouse gas emissions by “at least” 50% by 2050, compared with 2008 levels. This strategy is due to be revised by 2023 [56].

The EU MRV cumulative emissions implied by the IMO’s current global target are however far higher than a Paris compatible 1.5 °C budget. The IMO does not set out the rate at which emissions would fall by 2050 under its target, but it does have an interim 2030 target of an “at least” 40% cut in CO_2_ emissions per transport work by 2030. This implies flat emissions from now to 2030, given IMO expectations of growth in transport work, counteracting transport work efficiency savings [49]. With these assumptions, if emissions continued to fall post 2050 to zero by 2060 then the cumulative EU MRV emissions from 2019 would be 4150 MtCO_2_; if the zero-date was 2075, this value would be 4750. Both are considerably higher than the Paris-compatible range in Table 5 of 1070 to 2650 MtCO_2_.

The current IMO target gives much higher carbon budgets than Paris 1.5 °C carbon budgets for two reasons. First, the IPCC conclude that net zero emissions would be required by 2050 [57], not a reduction of 50%. Second, the lack of an absolute 2030 target means emissions reduction is delayed to later decades.

**Fig. 5:**
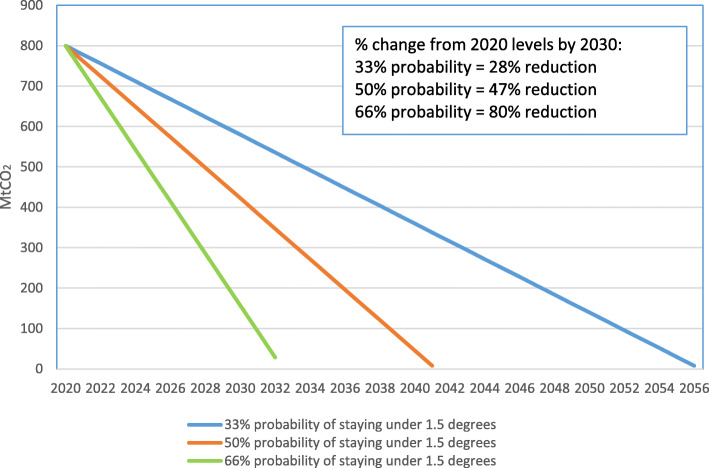
International shipping CO2 trajectories compatible with Paris 1.5°C target

Overall this implies that the IMO’s “at least 50%” target requires substantial tightening to be Paris-compatible. Using a proportionate-to-current-share approach, and the global carbon budgets set out in Table 5, linear reductions for international shipping to be Paris 1.5 °C compatible are set out in Fig. 5 below, with a central result of a required zero emission date of 2041, and a 47% cut in 2020 emissions by 2030. A sensitivity test of giving international shipping a 10% higher or lower share of the global carbon budget moves the zero emissions date only marginally, from 2041 to 2043 or 2039 respectively.

### Measures to reduce baseline committed emissions

Slower speeds, operational efficiencies and blended and zero-carbon fuels can lower baseline committed emissions without premature scrappage of existing ships. The values in Table 2 were inputted into the model (see supplementary information and accompanying spreadsheet), with results shown in Table 6.

**Table 6:** Effect of different measures on reducing committed emissions

	Inputs (Low-mid-high):			Committed emissions MtCO_2_ under different measures			% reduction on baseline committed emissions		
Individual measure:	Improvement	Start date	Years until fully deployed	Low	Mid	High	Low	Mid	High
Slow-speed	15–25%-35%	2030–2024-2022	10–5-3	2179	1919	1652	4	15	27
Technical and operational	5–20%-35%	2030–2025-2022	8–10-15	2190	1924	1629	3	15	28
Fuel blending	1% pa – 2% pa- 4% pa	2030–2025-2022	n/a	2256	2226	2048	0	2	9
Zero-carbon fuel	n/a	2035–2030-2025	15–10-10	2092	1722	1270	7	24	44
All 4 measures				1976	1320	793	13	42	65

These results show that there is potential for measures to reduce baseline committed emissions by 65%, to 793 MtCO_2_, assuming coverage of all four measures, with strong policies implemented rapidly. However, if these measures are implemented slowly, and to a lesser extent, then the reduction falls to just 13%, resulting in emissions of 1976 MtCO_2_. Applying any measure on its own also reduces potential emissions savings. Under the model’s mid-range assumptions, full retrofit of zero-carbon vessels within a decade from 2030 has the largest impact, but there are also large savings from speed reductions and from operational efficiencies in the 2020s.

A further analysis examined the effects of applying stronger policies, at mid-range timescales, versus mid-range policies, at faster timescales. It shows that implementing policies faster delivers greater savings than delivering stronger policies, although clearly doing both results in the highest reductions (Table 7).

**Table 7:** Effect on emissions reduction of varying the pace and strength of policy implementation

Policy strength	Policy implementation date	Committed emissions	% reduction on baseline committed emissions (2260 MtCO_2_)
Mid	Mid	1320	41
High	Mid	1115	51
Mid	High	949	58
High	High	793	65

Definitions for “mid” and “high” are as set out in Table 2

### New and replacement ships

Although there are measures which can reduce baseline committed emissions from existing ships, there are also committed emissions from future new ships. This section sets indicative values for the size of these additional committed emissions. New ships are split into two types: replacement ships for existing ships when they are scrapped, and “additional” ships reflecting the growth of shipping trade in total.

**Fig. 6:**
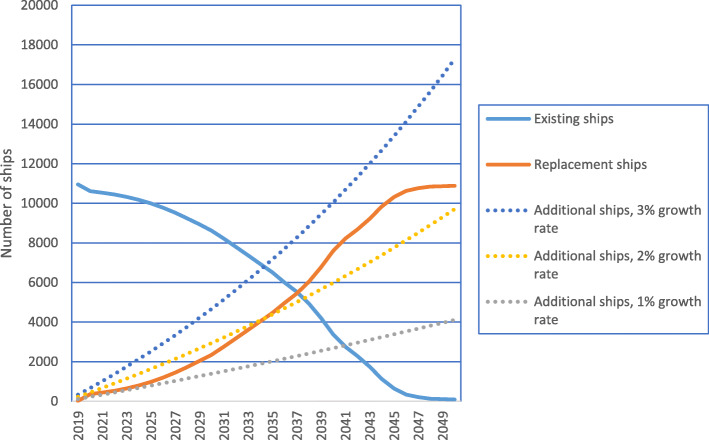
Number of ships in EU MRV under different assumptions of fleet growth rate

Figure 6 shows these three components of total fleet size under different assumptions for overall fleet growth.

Although in a high growth scenario the number of new additional ships dominates the total figure by 2050, in 2030 the majority of the shipping fleet is still existing ships for all assumptions on growth rate.

The replacement and additional new ships would contribute additional committed emissions. Assuming that these new ships have efficiencies 40% better than existing ships, and that there are further 3% annual EEDI improvements, then the new ship committed emissions before any additional operational efficiencies, fuel blending or slow steaming are set out in Table 8, for different assumptions on fleet growth and starting date for introduction of zero-carbon fuels.

**Table 8:** Effect of varying growth rate and year new ships start to use zero-carbon fuels on new ship committed emissions

	Annual growth rate of fleet:		
	1%	2%	3%
	Committed emissions (MtCO_2_):		
New ships start to use zero-carbon fuels from 2030, full deployment within 10 years	320	430	560
New ships start to use zero-carbon fuels from 2035, full deployment within 10 years	560	740	960

These results show the large difference in new ship committed emissions between a starting date of 2030 and 2035 for the adoption of zero-carbon ships and zero-carbon retrofits. A faster fleet growth rate also puts greater pressure on adoption of an early zero-carbon ship date to keep within carbon budgets. The combination of existing ships’ committed emissions (Table 6) and new ships’ committed emissions (Table 8) is shown in Table 9, compared with carbon budgets.

**Table 9:** Effect of varying zero-carbon ship date on committed emissions, versus carbon budgets

	Emissions in MtCO_2_				
	Existing ships	Replacement and additional ships	Total	Carbon budget for 50% 1.5 °C	Total as a % of a 50% 1.5 °C budget
Zero-carbon fuel ships available from 2030, fully deployed in 10 years, no operational/speed measures	1722	430	2152	1670	129%
Zero-carbon fuel ships available from 2035, fully deployed in 10 years, no operational/speed measures	2027	740	2767	1670	166%

Table 9 shows that zero-carbon fuels for all new ships from 2030, plus retrofitting of all existing ships over 2030–2040, still leaves shipping emissions over budget, at 2152 MtCO_2_ compared with a budget of 1670 MtCO_2_ for a 50% chance of exceeding 1.5 °C warming. However, other measures can also be applied. Using the mid-range input values for slow-speeds, technical and operational efficiencies and blended fuels in Table 2 would reduce existing ships’ committed emissions to 1320 MtCO_2_, a 400 MtCO_2_ saving. A similar percentage reduction for new ships would save a further 100 MtCO_2_, bring emissions in total down from 2152 to 1652 MtCO_2_.

## Discussion

There are three main contributions to the literature from this analysis, discussed in turn in this section. First, because ships have long lifetimes, the baseline committed emissions from existing ships are large: at 2260 MtCO_2_ this is 135% of the carbon budget for a 50% probability of exceeding 1.5 °C. New ships built in the 2020s will also add to this. Second, this committed emissions value could be considerably lower: if measures are introduced to lower ship speeds, improve operational efficiencies and use zero-carbon fuels it is possible for shipping to stay within a 1.5 carbon budget, the date of deployment being important. Third, there are significant differences in the age profile of different ship types, which has implications for decision-makers wanting to implement policies to cut shipping emissions. All three contributions highlight the importance of policies focussed on the existing fleet rather than solely on performance standards for new build such as the IMO’s EEDI.

### Shipping’s committed emissions

There are three previous papers that have considered shipping’s committed emissions, all of them treating shipping as a sub-set of transportation emissions as part of a global analysis, and all using extrapolations for shipping based on asset lifetimes elsewhere in the transport sector [12, 13, 23]. This paper adds significant value to the existing literature on committed emissions by calculating an in-depth committed emissions value in a large segment of the shipping sector, using for the first time shipping-sector-specific assumptions about asset lifetimes. The 2260 MtCO_2_ baseline committed emissions value in this paper is around twice what might be expected if following the Tong et al. [13] methodology, which is in turn based on Davis et al. [12]. The main cause of this difference is due to the different values used for the lifetimes of existing ships. This study uses an average of 28.3 years, whereas Tong et al. use assumptions for mean lives of motor vehicles, of 16.9–28.0 years. This paper’s value is similar to that derived by Smith et al. [23], who assume that ship lifetimes are similar to those of planes at 26 years. However, our use here of non-uniform age and lifetime profiles of different ship types and sub-types of ship is a further contribution.

The baseline committed emissions value for existing ships of 2260 MtCO_2_ is 135% of an EU MRV ships’ carbon budget for a 50% chance of staying below a 1.5 °C global temperature target (85 to 212% for 33 to 66% probability respectively). Global carbon budget values are subject to ongoing research, but the results of this analysis reinforce the finding of Tong et al. that committed emissions from existing high-carbon infrastructure leave very little room for new future high-carbon infrastructure. In addition, replacement and additional ships built in the 2020s will also run predominantly on fossil fuels, adding to committed emissions.

However, our larger committed emissions value could be lowered, if policies or practices are adopted that mean these long-lived assets use less or zero-carbon fuel in future.

### Lowering committed emissions below carbon budgets

Applying the findings of Bouman et al.’s 2017 review [30] of measures for reducing greenhouse gas emissions in shipping to latest individual ship level emissions data allows us to assess the sector’s compatibility with global climate targets and the balance between CO_2_ mitigation measures targeting the existing fleet and new builds.

The results show that even with rapid deployment of zero-carbon fuel in new ships from 2030 and if all the existing ships at that date were retrofitted to use zero-carbon fuels over the following 10 years, then the emissions from this fleet would be 2152 MtCO_2_, which is 129% of a carbon budget with 50% probability of 1.5 °C (See Table 9). If this date were delayed to 2035, committed emissions would total 2767 MtCO_2_, 166% of a 1.5 budget, highlighting the considerable additional emissions due to just a 5 year delay.

Assuming a 2030 zero carbon-fuel date, introducing additional measures such as lower speeds, fuel blending, and operational and technical efficiencies, based on mid-range assumptions from the literature, could lower emissions to 1652 MtCO_2_, just under a 50% 1.5 °C carbon budget.

Two points follow from this. First, it is imperative that zero-carbon fuels and associated infrastructure are developed and deployed at scale such that new ships running on such fuels are rapidly deployed from 2030 at the latest, and that existing ships are retrofitted from that date. Second, this is not sufficient for a 1.5 °C target, and slow-speed and efficiency measures also need to be deployed in the 2020s.

With an assumption of zero-carbon new ships from 2030, the vast majority of the total committed emissions comes from existing ships (80%), rather than ships built between now and 2030. This is because the turnover of the shipping fleet is slow: even with an assumption of annual fleet growth at 2%, older, less fuel-efficient ships dominate the fleet. With 80% of committed emissions coming from existing ships, this means that measures to cut emissions need to focus predominantly on these ships, rather than just measures such as the IMO’s EEDI policy [17], which focuses on new ships. A revised IMO Greenhouse Gas strategy should aim to reduce emissions in-line with carbon budgets for the Paris Agreement 1.5 °C goal. This paper suggests that for international shipping, a zero emission date around 2040 would be an appropriate goal, with an interim target of 47% cuts from 2020 emissions by 2030.

The analysis here shows that the individual measure with the greatest potential to deliver these emissions reductions is slower speeds, with major reductions from early implementation (Table 7). This adds further weight to the argument for adopting measures that incentivise or mandate slower ship speeds, as these could implemented far faster than the majority of the operational measures requiring retrofit in shipyards.

### Policy interventions and ship type

Analysis of emissions data by ship type can aid policy-makers by enabling a focus on areas with greatest potential. Container ships have the highest baseline committed emissions, at 29% of the total. The EU MRV data show that even though new container ships are among the most energy efficient vessels (in gCO_2_/t nm), the fact that they are so large, so new and so long-lived means they have a disproportionately large impact on total committed emissions. Paradoxically perhaps, this suggests a mitigation policy focus on the ships that are already some of the most efficient. Slow steaming and operational measures are well suited to bringing committed emissions down in the container ship type, reducing their committed emissions by 27% in this analysis. This is further reinforced by evidence that container ships on average travel at faster speeds than other ship types [49] meaning there is potentially great scope to reduce container emissions via this parameter.

Some types of ships are very long-lived, notably cruise, passenger and ro pax vessels. Slow steaming may be harder in these cases, which means that retrofit and operational efficiency measures are likely to bring the largest gains. Figure 2 shows a large range for average remaining life by ship type: for example refrigerated cargo ships have on average 9 years remaining life, whereas passenger ships have 29 years. All other things being equal, a ship owner will be less willing to invest in retrofitting measures to a ship with less remaining life. This means that for ships and ship types with shorter remaining life, the successful uptake of emissions reduction measures requires action from policy-makers, such as regulating on speed, retro-fitting to improve efficiency, or market-based mechanisms that impact on fuel price.

A further issue is the interplay between EU and non-EU markets. Refrigerated cargo vessels in the EU MRV are old, at 22 years on average, but outside the EU they are on average 9 years older. If retrofitting is not an economic option for old refrigerated cargo vessels, and they are sold on into non-EU markets, then the emissions would merely be transferred into other geographical areas. EU policy makers might therefore consider incentives for early scrappage for very inefficient older ships. The types where it appears that this might be more applicable are refrigerated cargo, container ships and oil tankers. Within ship types opportunities have also been identified. For example, compared to other ship types, container ships’ remaining lifespan is low, but within the container ship type, ships with shorter remaining life are smaller and less efficient. A differential approach might then be appropriate within the container fleet; speed reduction measures being appropriate for all ships, but with an additional emphasis on further operational measures for the larger, newer, more efficient ships, and early retirement for the older, smaller, less efficient ships.

Finally, a stated aim for the EU MRV system is that it helps to bring emissions down [26]. This initial analysis of the EU MRV suggests that its data is useful in highlighting to EU and global policy-makers which areas and policy interventions might deliver the largest emissions reductions benefits. The EU has also stated that it will take unilateral action on shipping’s CO_2_ emissions if there is insufficient progress at IMO level [58]. In such a situation the EU MRV dataset will be useful for policy makers and analysts in determining priority areas for climate mitigation measures in the shipping sector. It is intended that the EU MRV programme should be integrated with the global IMO Data Collection System (DCS) over time. To aid policy-makers, industry and others in determining effective interventions, we suggest that the IMO should similarly make individual ship data from their forthcoming DCS monitoring programme publicly available, as is the case with EU MRV.

## Conclusions

This study provides a new assessment of the scale of the mitigation agenda for the shipping sector, and the imperative for significantly accelerating efforts to target CO_2_ policies within the existing global fleet. Building on Tong et al. [13], we analyse recently published EU ship CO_2_ data, with shipping sector-specific data for ship lifetimes and find committed emissions for this EU dataset to be twice that presented in their study. We find that existing ships are expected to contribute 85 to 212% of the sub-sector’s 1.5 °C-compatible carbon budget. Emissions from replacement and additional ships in the 2020s would add to this, further exhausting carbon budgets.

The ships that were in operation at the time of writing in early 2020 will still make up the majority of the fleet in 2030. To keep within carbon budgets, the shipping sector will need not only to adopt new very low-carbon fuels and new very low-carbon ships from 2030 at the latest, but in addition rapidly deploy measures such as operational efficiency and impose slower speeds *within* the 2020s to mitigate the committed emissions from the existing fleet. Combining these measures could cut the baseline committed cumulative CO_2_ emissions from existing ships by up to 65%. Comprehensive adoption of the mid-range assumptions (Table 2) for existing and new ships would be sufficient to stay within Paris compatible carbon budgets.

The shipping sector has a broad suite of options to decarbonise but needs major policy interventions to incentivise change to the existing fleet. By distinguishing between types of ship, this analysis illustrates the huge value of retrofit solutions that help shipping align with Paris goals. Specifically, container ships are shown to be the greatest contributor to committed emissions, and perhaps counter-intuitively, newer ships are the biggest contributors within the container category, despite being more efficient because they tend to be larger in size. Passenger ships have disproportionately high committed emissions, given their small numbers, because they tend to be very long-lived: regulation that improves operational efficiencies should therefore be a priority in this type of ship. Across ship types, slow steaming stands out as the most promising measure that could be applied quickly to deliver large reductions in emissions in line with Paris goals.

The committed emissions from ships are significant, yet a combination of policies on very low-carbon ships from 2030, combined with speed and operational measures from the early 2020s, could keep shipping within a Paris-compatible carbon budget. However, any delay to appropriate policy implementation would mean additional measures, including demand-side or early scrappage interventions, to meet the Paris climate goals. In summary, the time left to deliver on what is dictated by the global Paris Agreement is too short to rely on measures that predominantly focus on improving the efficiency of new ships. New zero-carbon ships are essential, but we conclude that policy makers must target a new suite of mitigation measures at the existing fleet with some urgency.

## Supplementary information

**Additional file 1.**

**Additional file 2.**

## Data Availability

Raw EU MRV data is available from https://mrv.emsa.europa.eu/ Raw data on ship age and lifetimes was taken from Clarksons World Fleet Register and World Shipyard Monitor; this data is commercially confidential and cannot be disclosed here. Supplementary information contains a file with aggregate analysis of EU MRV and Clarksons data, and model inputs and results.

## Abbreviations

CO_2_: Carbon dioxide
DCS: Data Collection System
DWT: Deadweight Tonnage
EEDI: Energy Efficiency Design Index
EU MRV: European Union Monitoring, Reporting and Verification Scheme
IMO: International Maritime Organization
IPCC: Intergovernmental Panel on Climate Change
LNG: Liquefied Natural Gas
Mt: Million tonnes
tnm: Tonne-nautical mile
