# Supplementary material for: Shipping and the Paris climate agreement: a focus on committed emissions
Source: BMC Energy. 2020 Jun 12;2(1):5. doi: 10.1186/s42500-020-00015-2 (PMC8824533; doi:10.1186/s42500-020-00015-2)
Supplement: Supplementary file 1 — Additional file 1. [file 42500_2020_15_MOESM1_ESM.pdf]

Shipping and the Paris climate agreement: a focus on committed emissions

Supplementary information file

This section contains additional information on methodology. It covers four issues: i) data quality; ii) calculating baseline committed emissions; iii) measures to reduce baseline committed emissions; and iv) EU versus global carbon budgets. There is an accompanying spreadsheet showing calculations and inputs regarding issues ii) and iii).

Data quality

There is very high congruence between Clarksons and EU data, by matching ship name to ship IMO number across the two datasets. There are around 20 ships (0.18%) in the EU set not in Clarksons – checking against a third source (fleetmon.com) shows that the reason for all but one of these is that they have been put out of service since the EU data was published[1]. These 20 ships are not included in the calculations for baseline committed emissions.

There are very few outlier values in the EU CO_2 data. The majority appear to be recording errors. The EU says it is not responsible for any errors, and these should be brought up with the relevant ship data verifier[2]. Some five instances were clear errors (e.g. a value several orders of magnitude higher than every other vessel) so these ships were removed from the analysis. There was also a persistent occasional error where the ratio of fuel to CO_2 for a ship was more than double that for every other ship in that type. Again this was assumed to be a simple verifier error, affecting 20 ships, and the CO_2 value was corrected so that it is the same ratio to fuel consumption as every other ship in that type. In total, these errors account for less than 0.2% of the fleet. Finally, 597 ships have no CO_2/fuel data – these ships are simply not used in the analysis.

Table S1: Number of ships in EU MRV database compared with EU and Global totals (1, 2)

Number of ships	In the EU MRV system	Total (including smaller ships)
EU ships	11,566	21,029
	Global	Total
Global ships of the same type	64,029	97,645
EU as % of global	18%	22%

Source: EU and Clarksons data (1, 2)

[1] Third source was www.fleetmon.com. EU data covers the year 2018; Clarksons data is for September 2019.
[2] Personal communication with EU MRV staff

The EUMRV data set is new, and there is not yet detailed analysis in the literature of the reliability of this data. Panagakos et al (3) discuss aspects of the data set in detail, and are for example critical about the validity of some of the EUMRV's ship efficiency data. However, the values for these efficiency indicators are by definition more subject to uncertainty than simple fuel consumption data: for example an efficiency measure of fuel consumption/tonne-mile is product of fuel consumption, distance, and cargo – tripling the sources of potential uncertainty compared with simple reporting on fuel consumption. In our study, the focus is on absolute CO_2 emissions, a direct product of fuel consumption and standard fuel emission factors, so this data is likely to be more reliable than for efficiency measures. However, data quality is an ongoing issue for EUMRV and subsequent analyses of it, and we note Panagakos' suggestions for improvements, for example addressing the issue that reporting obligations fall to ship owners rather than ship operators.

Calculating baseline committed emissions

The baseline committed emissions of an individual ship, $E(i)^{ship}_{baseline}$, across its remaining lifetime with no carbon reduction measures applied, is calculated by multiplying the ship's future life in years by its annual emissions in 2018, $E(i)^{ship}_{annual}$ (equation 1). A ship's future life is calculated by subtracting its current age, $t(i)_{ship\ age}$, from the average age at scrappage for that ship's type and size, $t_{scrappage}$. For some ships this gives a negative value – ie ships who are already older than the fleet average scrappage age. In this calculation, these ships are assumed to be scrapped next year. This assumption will therefore underestimate committed emissions, as not all of these very old ships will be scrapped next year, however their expected lifetime is sufficiently low, and the number of ships it affects (0.7%) means this assumption will have a far lower than 1% impact on the final value.

The total baseline emissions from each ship class, $E(i)^{class}_{baseline}$, is calculated by summing across the total number of ships in each class, N_T, where i represents each individual ship. The total baseline emissions from the full fleet, $E\ ^{fleet}_{baseline}$, is then calculated by summing across the total number of classes, N_{class}.

$$E(i)^{ship}_{baseline} = [t(i)_{ship\ age} - t_{scrappage}] * E(i)^{ship}_{annual} \quad 1$$

$$E(i)^{class}_{baseline} = \sum_{i=1}^{N_T} E(i)^{ship}_{baseline} \quad 2$$

$$E(i)^{fleet}_{baseline} = \sum_{class=1}^{N_{class}} E(i)^{class}_{baseline} \quad 3$$

The mean age a ship is scrapped is estimated, by taking the values of average scrappage age for each ship type and sub-type, for each year in the last ten years' publications of Clarkson's World Shipyard Monitor (WSM), and taking an average value across these ten years. Compared with the 2018 value the overall ten-year average value is approximately 5% lower (see Figure S1), however, ten-year averages were deemed more appropriate given the short-term cyclical nature of shipping scrappage markets (4).

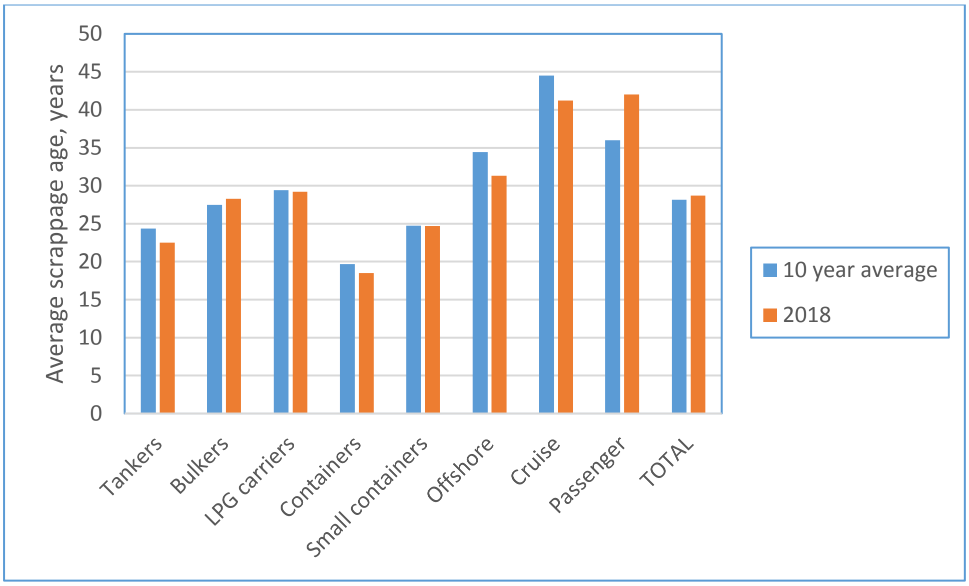

Figure S1: Average scrappage age by ship type, 10 year average (2009-2018) vs 2018 value. Source: Clarksons World Shipyard Monitor, multiple years.

Clarkson's World Fleet Register contains values for the current age of every ship. This database is merged with the EU MRV database and the WSM average scrappage age results to give a value for the average predicted remaining life for each ship in the EU MRV database, referencing against each ship's IMO number.

A baseline committed emissions value for each ship is then calculated using the equations above, assuming each ship continues to be used at the same rate as in 2018, with the same level of annual emissions. This assumption, that newer ships are not used more frequently or used on longer routes than older ships, cannot be verified directly, however, analysis of fleet time-at-sea data offers some insight (Figure S2). Correlation between ship age and time at sea is a statistically significant but very weak positive relationship (Spearman's rho = 0.19, $p < 0.0005$ two tailed). Further analyses were also conducted for all 14 ship types, and sub-divisions within them (for example ships only undertaking non-EU-EU journeys and only undertaking intra-EU journeys), with similar results. However, there are two major caveats over the use of this time-at-sea vs age data to infer potential different assumptions around emissions over time. First, this insight is of new ships now versus old ships now, rather than of a ship's emissions profile over time. For this we would need multiple years' EU MRV data. Second, this data does not include time at sea for journeys which do not involve any EU port. To get accurate time at sea vs age data we would need a global system. Here then we acknowledge that the emissions profile over time is currently an unknown, which will only become clear with a global monitoring and reporting system with multiple years of data. For now, this study assumes constant emissions over time for a given ship.

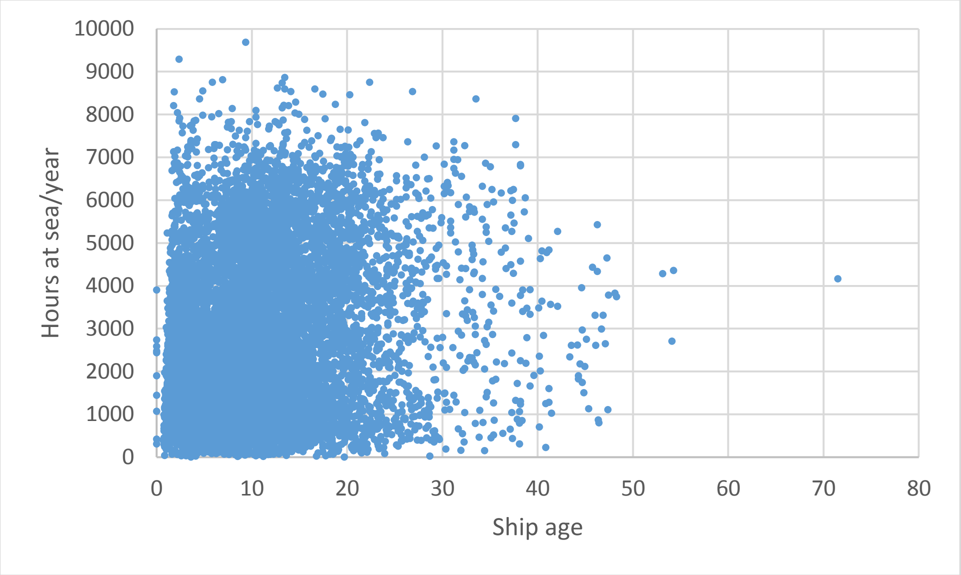

Figure S2: EU MRV fleet, age vs time at sea.

Matching the age data with ship size data highlights variations within ship types. For example, within the container ship type it is newer container ships that are responsible for the highest committed emissions (Figure S3). Despite newer container ships being much more efficient (Figure S4, Spearman's correlation coefficient rho = -0.567, $p < 0.001$), the recent trend for container ships to become much larger (Figure S5, Spearman's correlation coefficient rho = -0.575, $p < 0.001$) has a greater impact.

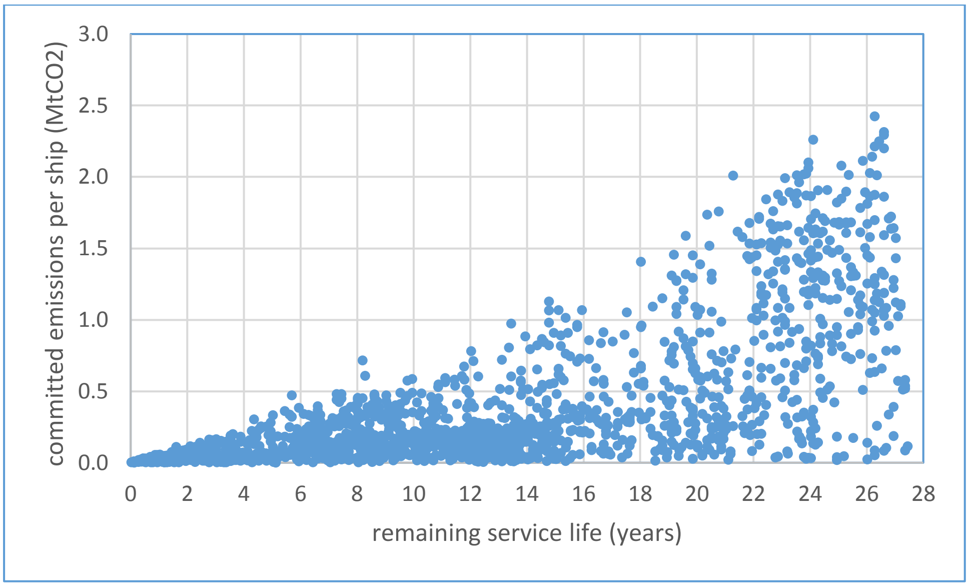

Figure S3: Container ship committed emissions vs remaining service life

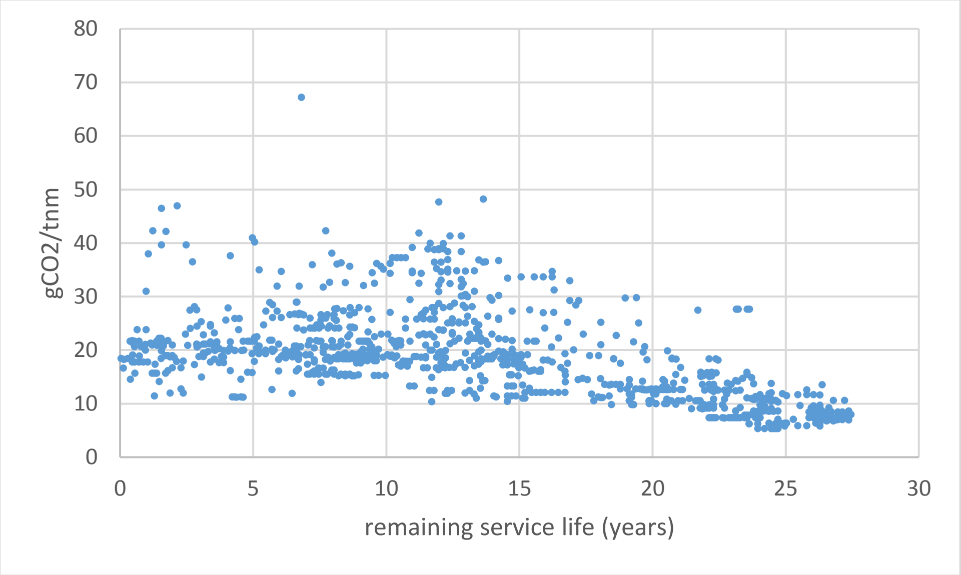

Figure S4: Ship technical efficiency (gCO_2/t nm) vs years remaining service life, container ships

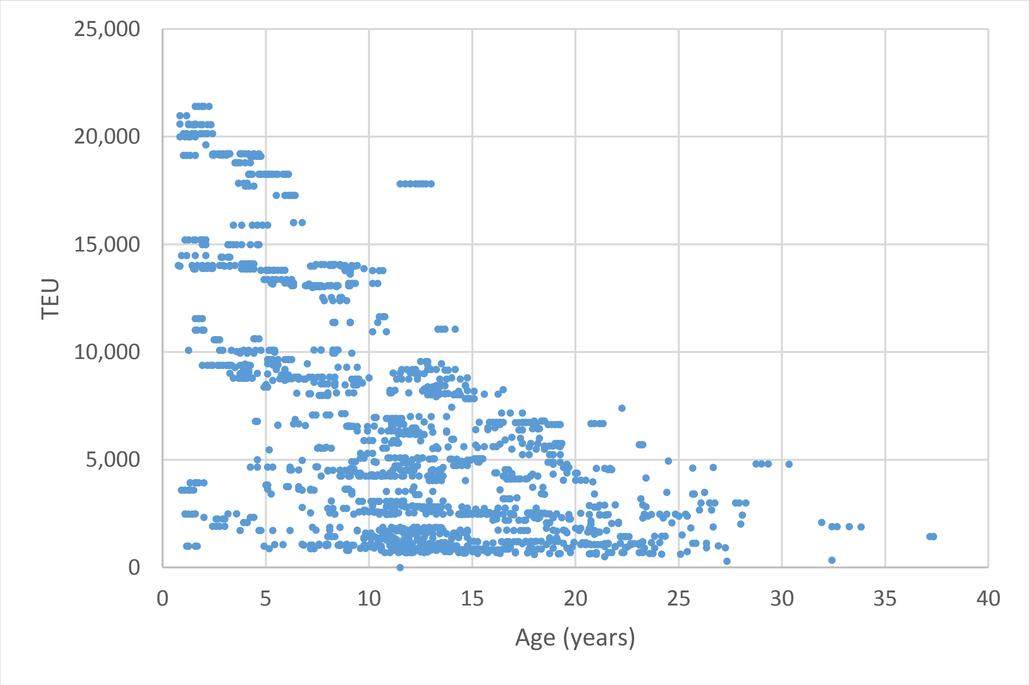

Figure S5: EU container fleet: size in TEU compared with age in years

Measures to reduce baseline committed emissions

In the model constructed for this paper, four types of measure were applied sequentially to reduce baseline committed emissions: slower speeds, operational improvements, blended fuels, and zero-carbon fuels. This section sets out the assumptions underpinning the values used for each of these

measures (see Table 2), the equations for how each measure individually affects the baseline emissions value, and the equations for how the four measures are applied sequentially.

The spreadsheet accompanying this paper allows users to alter their chosen input parameters for these four measures for each ship type.

The assumptions behind the values set out in Table 2 are:

Slow speed

The effect of speed reductions on fuel consumption is complex. The power required for a ship's propulsion is proportional to speed cubed (5). But if speed is lowered, that power is required for longer to cover the same distance, so energy saving at lower speed is less than a cubic factor. In addition, for each ship there are different optimal speeds for efficient engine operation, and for a given value of transport demand, there are potential rebound effects due to the need for more ships to transport the same quantity of goods in the same time (6). Overall the literature cites large potential savings in fuel consumption from lower speeds, but with wide ranges: the review by Bouman et al. (7) cites savings from 1-60%, depending on assumptions, with a range of 15-35% between first and third quartiles, which is used here, similar to the range of 13-33% cited by Faber et al. (8). Slow steaming has been occurring to some extent already, and it is uncertain how much further reduction is possible. Rutherford et al. (9) show that the engine operating power of container ships, bulk carriers and tankers in 2018 were largely between 20-50% of an engine's Maximum Continuous Rating (MCR). Therefore, in some cases, achieving high savings from speed reduction would require innovative solutions, such as engine de-rating (10).

Speed reductions can occur voluntarily, and can make sense when fuel prices are high, but here it is assumed that widespread and long-lasting uptake speed reductions would require regulation, agreed at IMO level. This issue is on the table at IMO Marine Environment Protection Committee (MEPC) meetings, however progress has recently stalled (11). A minimum of three and a maximum of ten years before regulations would be enacted is assumed here, and three to ten years before these are fully applied. In addition, sensitivity analysis were performed to understand the effects of non-application for particular ship classes, where speed restrictions may be more problematic. As an example, assuming that Ro Pax/Passenger/Reefer had speed factor improvement of 1 instead of 0.75 (mid) increases their committed emissions by 26%, but increases total committed emissions for all ships by just 5%.

This analysis integrates emission reduction measures by applying an emission reduction factor to the *annual* baseline emissions from each ship class. The annual baseline committed emissions from a class, $E(t)^{class}_{annual}$, is calculated by summing across the annual baseline committed emissions of each individual ship of that ship class.

$$E(t)^{class}_{annual} = \sum_{i=1}^{N} E(t,i)^{ship}_{annual} \qquad 4$$

Where

$$E(t,i)^{ship}_{annual} = \begin{cases} 0, & t_{ship\ age} \geq t_{scrappage} \\ E(i)^{ship}_{annual}, & t_{ship\ age} < t_{scrappage} \end{cases} \qquad 5$$

The total committed emissions for each class including speed reduction, E^{class}_{speed}, is then calculated by applying a speed reduction factor to the classes annual emissions each year, $E(t)^{class}_{annual}$, and then

summing the reduced annual emissions across the total number of years, from a start year of 2019 (t_0) to an end year of 2060 (t_{end}). The calculation assumes a start year for speed reductions as t_{start}, the percentage number of ships implementing speed reduction of $N_s(t)$, an emission reduction factor for speed reduction, f_s, and annual baseline emissions, $E(t)^{class}_{annual}$ (equation 6). $N_s(t)$ is a function of time and increases from 0 to 1 from a start year of t_{start} to a year of full fleet implementation, t_{full}, which are both set manually. $N_s(t)$, therefore, depends on the number of years taken to achieve fleet-wide implementation of speed reduction, assuming a linear year-on-year adoption rate.

$$E(t)^{class}_{speed} = \sum_{t_0}^{t_{start}} E(t)^{class}_{annual} + \sum_{t_{start}}^{t_{end}} E(t)^{class}_{annual}(1 - N_s(t) f_s) \qquad 6$$

Where

$$N_s(t) = \begin{cases} 0 < N_s(t) < 1, & t_{start} < t < t_{end} \\ 1, & t \geq t_{end} \end{cases} \qquad 7$$

Technical and Operational factors

A very wide variety of operational measures could be applied, such as voyage optimisation, the use of wind-power (rotors, sails), shore power, and resistance reduction devices. The evidence from Bouman et al. and other sources (7, 12) suggest that these mostly operate in the 0-10% improvement range, and not all are applicable to every vessel type – for example Flettner rotors are more appropriate for bulk carriers than for containers. There would also be a lag in deployment. For example, some improvements require retrofits in shipyards, and capacity is limited: the current rush to fit scrubbers to existing ships to meet the 2020 IMO air pollution regulations has seen just 3,000 vessels retrofitted(13). We assume that overall, the sum of operational measures could deliver 5-35% improvements across the fleet, with deployment taking 8-15 years.

The committed emissions including technical and operational measures are calculated through the same process as for speed reduction, but replacing the emission reduction factor for speed, f_s, with the reduction factor for technical/operational measures, f_t, and using updated assumptions for t_{start} and $N_t(t)$.

Blended fuels

There is the potential for ships to use a proportion of zero-carbon fuel in their engines, and there are uncertainties over which zero-carbon fuels will dominate in the long-term. Shipping companies are starting to explore the use of biofuels, for example Maersk recently trialling the use of up to 20% blended biofuels on a containership journey from Rotterdam to Shanghai (14). Use of blended fuel is constrained however by cost, scalability and the uncertainties over whether fuels are genuinely low or zero-carbon, for example biofuels (15). On biofuel carbon emissions, the literature shows wide ranges in greenhouse gas emissions (16), for example between –634 and +260 kgCO2/MWh for UK bioenergy sources (17). On scalability similarly, there are very large ranges for the potential global bioenergy resource (18), eg 22-1272 EJ (19), let alone the proportion of this which could be realised without negative impacts on other sustainability goals. Any use of biofuels in shipping would need strict sustainability standards to ensure genuine emissions savings, and to ensure its production was not in conflict with other sustainability goals. Here, it is assumed that blending will become more commonplace in the 2020s, with ships using blended fuels increasing 2-4% a year, and the proportion of blended fuel also increasing at 2-4% a year, and this use of biofuels would deliver 100% emissions savings over use of conventional diesel fuel.

This study assumes that ships increase the amount that their fuels are blended by δ each year, with the percentage number of ships using blended fuel increasing by ΔN_b per year. Therefore, in the nth year, the committed emissions from ships *not* using blended fuel is

$$E(t)_{no\ blend} = E(t)^{class}_{annual}(1 - \Delta N_b n) \qquad 8$$

As ships percentage fuel blend increases by δ each year, in the nth year ships using blended fuel will produce annual emissions of $E(t)^{class}_{annual}(1 - \delta n)$. However, as the total number of ships using blended fuels increases by ΔN_b each year, the committed emissions from ships using blended fuels in the nth year is

$$E(t)_{blend} = E(t)^{class}_{annual}\left[\Delta N_b(1 - \delta n) + \Delta N_b\big(1 - \delta(n-1)\big) + \Delta N_b\big(1 - \delta(n-2)\big) + \cdots\right] \qquad 9$$

Simplifying this, equation 9 becomes

$$E(t)_{blend} = E(t)^{class}_{annual}\Delta N_b[n - \delta(n + (n-1) + (n-2) + \cdots)] \qquad 10$$

As $\sum(1 + 2 + \cdots + N) = {}^{N(N+1)}/_{2}$, equation 10 becomes

$$E(t)_{blend} = E(t)^{class}_{annual}\Delta \mathrm{N_b}\left[n - \delta\frac{n(n+1)}{2}\right] = E(t)^{class}_{annual}\Delta N_b n\left[1 - \delta\frac{(n+1)}{2}\right] \qquad 11$$

Using the above equations, the committed emissions for implementing blended fuels is calculated, $E(t)^{class}_{blend}$, assuming an implementation year of t_{start}, with the percentage number of ships using blended fuels of $N_b(t)$ each year and the proportion of blended fuel increasing by δ per year.

$$E(t)^{class}_{blend} = \sum_{t_0}^{t_{start}} E(t)^{class}_{annual} + \sum_{t_{start}}^{t_{end}} E(t)_{no\ blend} + E(t)_{blend} \qquad 12$$

Combining equations 8, 11 and 12 gives

$$E(t)^{class}_{blend} = \sum_{t_0}^{t_{start}} E(t)^{class}_{annual} + \sum_{t_{start}}^{t_{end}} E(t)^{class}_{annual}\left[1 - \Delta N_b(t - t_{start})\frac{\delta(t - t_{start}) + 1}{2}\right] \qquad 13$$

As ΔN_b is the percentage *increase* in the number of ships using blended fuels each year, then the percentage *total* number of ships using blended fuels in that year, $N_b(t) = \Delta N_b(t - t_{start})$, where $N_b(t)$ is subject to the same constraints as equation 7. Therefore,

$$E(t)^{class}_{blend} = \sum_{t_0}^{t_{start}} E(t)^{class}_{annual} + \sum_{t_{start}}^{t_{end}} E(t)^{class}_{annual}\left[1 - N_b(t)\frac{\delta(t - t_{start}) + 1}{2}\right] \qquad 14$$

$$E(t)^{class}_{blend} = \sum_{t_0}^{t_{start}} E(t)^{class}_{annual} + \sum_{t_{start}}^{t_{end}} E(t)^{class}_{annual}[1 - N_b(t)f_b] \qquad 15$$

Where

$$f_b = \frac{\delta(t - t_{start}) + 1}{2} \qquad 16$$

LNG is a fuel which can reduce local NOx and SOx air pollution. It can also have lower CO_2 emissions than diesel fuel, although the extent of this reduction is uncertain, given issues around methane slippage (20). Even if slippage were not an issue, there are still considerable lifecycle LNG GHG

emissions and Gilbert et al. (15) conclude that without on-board CCS, there is "*limited opportunity to reduce GHG emissions from LNG".* The use of LNG is growing, but it should not be seen as solution to shipping's CO_2 mitigation challenge. Shipping, like all sectors, will need to be zero carbon by 2050 to meet the Paris 1.5 degree goal (21). As ships have an average lifetime of 28 years, ships built to use LNG in the early 2020s will need to be subsequently retrofitted to use genuinely zero carbon fuels.

Zero-carbon fuel retrofits

Zero-carbon energy for ships could come in a number of forms – chiefly ammonia, hydrogen, and batteries, amongst others. There are uncertainties about scalability, cost, range, and carbon benefits for these alternative fuels (22, 23). In addition, large-scale deployment of any options may take many years, for instance DNV-GL note that it has taken LNG 20 years to reach 1% global deployment (24). A global consortium of 60 companies have set a goal of deployment of zero-carbon vessels by 2030 (25). Earlier dates may be possible – for example the UK Government has set a similar goal for 2025 (26). In this study we assume a range of 2025-2035 for deployment of zero-carbon vessels, and a corresponding date range for the start of conversion of existing ships to use zero-carbon fuels. This transition may take many years – it is assumed here that the imperative of climate change would drive full deployment within 10-15 years.

This study calculates the committed emissions including the uptake of zero-carbon fuels, $E(t)_{zc}^{class}$, assuming the percentage number of the fleet using zero carbon fuels increases by $N_{zc}(t)$ per year.

$$E(t)_{zc}^{class} = \sum_{t_0}^{t_{start}} E(t)_{annual}^{class} + \sum_{t_{start}}^{t_{full}} E(t)_{annual}^{class}\big(1 - N_{zc}(t)\big) \qquad 17$$

Combining emission reduction measures

When multiple measures are applied in a single year, the committed emissions for that year, $E(t)_{combined}^{class}$, is calculated by applying the measures sequentially.

$$E(t)_{combined}^{class} = E(t)_{annual}^{class}(1 - N_s(t)f_s) * (1 - N_t(t)f_t) * (1 - N_b(t)f_b\ ) * \big(1 - N_{zc}(t)\big) \qquad 18$$

The committed emissions from that ship class, $E\,_{reduced}^{class}$, is calculated by combining all emission reduction measures with specified start dates and uptake rates for each measure.

$$E\,_{reduced}^{class} = \sum_{t_0}^{t_{end}} E(t)_{combined}^{class} \qquad 19$$

The committed emissions from all ships, $E\,_{reduced}^{fleet}$, is then calculated by summing this across the total number of classes, N_{class} (equation 20).

$$E\,_{reduced}^{fleet} = \sum_{class=1}^{N_{class}} E\,_{reduced}^{class} \qquad 20$$

These variables, equations and calculations can be seen in the sheets for each ship class, cells B15 to AT34, of the accompanying spreadsheet. The summed values across all classes can be seen in sheet "MASTER INPUT", cells C15 to R20.

EU vs Global carbon budgets

The carbon budget for the ships covered in the EU MRV is calculated from the range of the appropriate global budget and compared with the committed emissions values (see results section).

The analysis in this paper calculates committed emissions and a carbon budget solely for the ships covered by the EU MRV budget. It does not use the EU MRV data to estimate a committed emissions value for the global fleet. This is because there are significant differences within ship types between the characteristics of the ships in the EU MRV and their equivalents at a global level. For this paper we analysed the differences between EU and global data for two ship types – containers and refrigerated cargo vessels. For example, for containers, ships in the EU MRV are on average much larger than the global average, as shown in Figure S6.

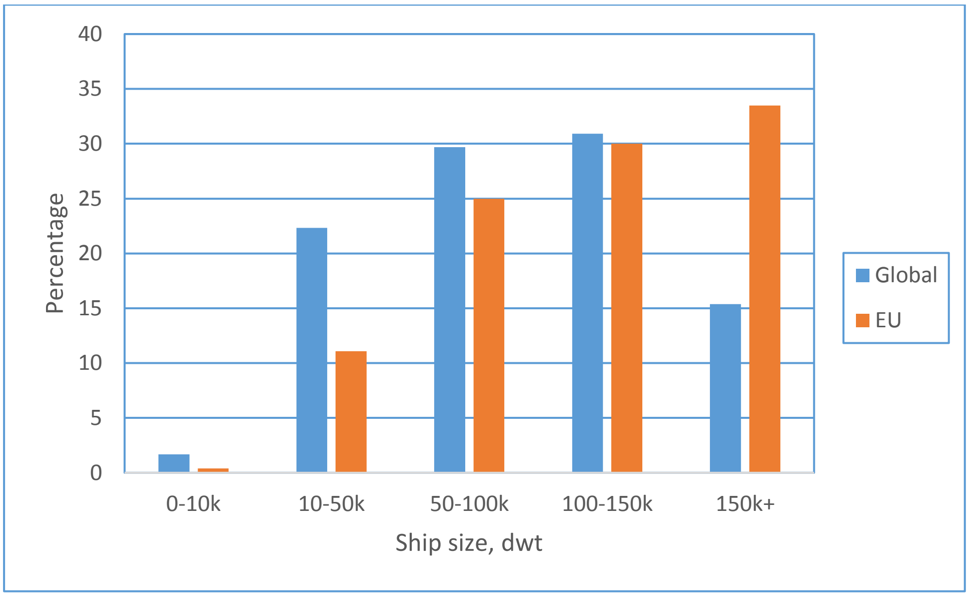

Figure S6: Percentage of container fleet tonnage in each size band, global vs EU MRV ships

For refrigerated cargo ships, EU MRV ships are on average three times larger and nine years younger than the global average. These results suggest it would not be appropriate to assume that analysis at an EU level could be translated up to the global level. Similar conclusions about the non-transferability of the EU MRV data to the global level are made by Panagakos et al. (3) in their paper on bulk carrier MRV data, with their analysis that Energy Efficiency Operational Indicator (EEOI) values are lower for ships in the EU MRV, compared with globally.

Supplementary references

1. EU Parliament. Regulation (EU) 2015/757 of the European Parliament and of the Council of 29 April 2015 on the monitoring, reporting and verification of carbon dioxide emissions from maritime transport, and amending Directive 2009/16/EC. 2015.
2. Clarksons. World Shipyard Monitor. 2019.
3. Panagakos G, Pessôa TdS, Dessypris N, Barfod MB, Psaraftis HN. Monitoring the carbon footprint of dry bulk shipping in the EU: An early assessment of the MRV regulation. Sustainability. 2019;11(18):5133.
4. Stopford M. Maritime economics 3rd edition: Routledge; 2009.

5. Lindstad HE, Eskeland GS, Rialland A. Batteries in offshore support vessels–Pollution, climate impact and economics. Transportation Research Part D: Transport and Environment. 2017;50:409-17.
6. Smith TWP. Technical energy efficiency, its interaction with optimal operating speeds and the implications for the management of shipping's carbon emissions. Carbon Management. 2012;3(6):589-600.
7. Bouman EA, Lindstad E, Rialland AI, Strømman AH. State-of-the-art technologies, measures, and potential for reducing GHG emissions from shipping–a review. Transportation Research Part D: Transport and Environment. 2017;52:408-21.
8. Faber J, Huigen T, Nelissen D. Regulating speed: a short-term measure to reduce maritime GHG emissions. CE Delft; 2017.
9. Rutherford D, Mao X, Osipova L, Comer B. Limiting engine power to reduce CO_2 emissions from existing ships. INTERNATIONAL COUNCIL ON CLEAN TRANSPORTATION; 2020.
10. Rehmatulla N, Calleya J, Smith T. The implementation of technical energy efficiency and CO2 emission reduction measures in shipping. Ocean engineering. 2017;139:184-97.
11. Marine Log. IMO panel backs away from ship speed or power limits. November 15th 2019 [Available from: https://www.marinelog.com/news/imo-panel-backs-away-from-ship-speed-or-power-limits/.
12. DNV GL. Maritime forecast to 2050. Energy Transition Outlook 2018. 2018.
13. Clarksons. Weekly Scrubber Update. Accessed 9th December 2019. 2019.
14. Maersk partners with global companies to trial biofuel. 22nd March [press release]. 2019.
15. Gilbert P, Walsh C, Traut M, Kesieme U, Pazouki K, Murphy A. Assessment of full life-cycle air emissions of alternative shipping fuels. Journal of Cleaner Production. 2018;172:855-66.
16. Gough C, Garcia-Freites S, Jones C, Mander S, Moore B, Pereira C, et al. Challenges to the use of BECCS as a keystone technology in pursuit of 1.5 ^{o}C. Global Sustainability. 2018;1.
17. Welfle A, Gilbert P, Thornley P, Stephenson A. Generating low-carbon heat from biomass: life cycle assessment of bioenergy scenarios. Journal of Cleaner Production. 2017;149:448-60.
18. Slade R, Saunders R, Gross R, Bauen A. Energy from biomass: the size of the global resource. Imperial College Centre for Energy Policy and Technology and UK Energy Research Centre, London.; 2011.
19. Slade R, Bauen A, Gross R. Global bioenergy resources. Nature Climate Change. 2014;4(2):99-105.
20. Ushakov S, Stenersen D, Einang PM. Methane slip from gas fuelled ships: A comprehensive summary based on measurement data. Journal of Marine Science and Technology. 2019:1-18.
21. IPCC. Summary for Policymakers. In: Global Warming of 1.5°C. An IPCC Special Report on the impacts of global warming of 1.5°C above pre-industrial levels and related global greenhouse gas emission pathways, in the context of strengthening the global response to the threat of climate change, sustainable development, and efforts to eradicate poverty. In: Masson-Delmotte V, P. Zhai, H.-O. Pörtner, D. Roberts, J. Skea, P.R. Shukla, A. Pirani, W. Moufouma-Okia, C. Péan, R. Pidcock, S. Connors, J.B.R. Matthews, Y. Chen, X. Zhou, M.I. Gomis, E. Lonnoy, T. Maycock, M. Tignor, and T. Waterfield (eds.), editor. 2018.
22. Lloyd's Register. Zero-emission vessels: transition pathways. 2019.
23. Ryste J. Comparison of alternative marine fuels - options and limitations. Presentation to Alternative Fuels Online Conference. September 25t^{h}. DNV GL; 2019.
24. Longva T, editor Maritime Forecast to 2050 - insights from the third edition. Presentation to Alternative Fuels Online Conference. September 25t^{h}2019: DNV GL.
25. Reuters. Shipping sector sets course for zero carbon vessels, fuel by 2030. 23rd September 2019.
26. Department for Transport. Clean maritime revolution starts voyage. 2018.
